# Differences and Similarities in Diabetes Research between China and the USA

**DOI:** 10.3390/ijerph16162989

**Published:** 2019-08-20

**Authors:** Hong Fan, Sheetal Bhurke, Guizhen Jia, Fujian Song

**Affiliations:** 1 Department of Social Medicine and Health Education, School of Public Health, Nanjing Medical University, Nanjing 211166, China; 2 National Institute for Health Research (NIHR), Evaluation, Trials and Studies Coordinating Centre (NETSCC), University of Southampton Beta House, Southampton SO16 7NS, UK; 3 Nanjing Medical University, Nanjing 210008, China; 4 Faculty of Medicine & Health Science, University of East Anglia, Norwich NR4 7TJ, UK

**Keywords:** non-communicable disease, diabetes mellitus, comparative study, China, USA

## Abstract

Diabetes mellitus is one of the major non-communicable diseases (NCD) with increasing prevalence in China. There is a lack of high-quality research focusing on prevention and management of diabetes in low and middle income countries (LMICs) compared to developed countries. This comparative study aims to describe the characteristics of diabetes research conducted in China and the USA. The study included 800 studies on diabetes mellitus from both countries. Compared with studies in the USA, studies in China were more likely to be laboratory-based primary research (50.5% versus 30.8%), more likely to use animal subjects (47% versus 27.5%), more likely to focused on risk factors (22.7% versus. 14.7%), more likely to be case-controlled studies (17.7% versus 10.0%), and more likely to evaluate pharmacological treatments (36.5% versus 20.7%). Further, compared with studies in the USA, studies in China were less likely to involve patients (42.7% versus 60.7%), less likely to be clinical trials (6.2% versus 14.5%), less likely to be cohort studies (8.8% versus. 26.0%), and less likely to evaluate disease management interventions (3.3% versus 13.3%). Clinical studies in China should be more patient-based to facilitate more effective control and management of diabetes.

## 1. Introduction

The burden of non-communicable diseases (NCDs) is rapidly increasing in low and middle income countries (LMICs). NCDs are already the main causes of deaths and disabilities globally [1]. The United Nations has recommended a shift in healthcare priority from infectious diseases to NCDs [2]. Clinical and public health decision-making needs to be informed by research regarding the burden of NCDs, risk factors, genetic variation, influence of socio-economic changes, effects of interventions, health system changes required, and implementation of the research in practice [3]. 

Studies on NCDs from LMICs is limited [4,5,6] and of poorer quality than those conducted in developed countries [7]. For example, a bibliometric analysis of diabetes research in India showed that research was focused more on epidemiology and less on prevention strategies or therapeutic interventions [8]. 

Diabetes mellitus is one of the major NCDs with high and increasing prevalence in China [9]. The prevalence of diabetes in China has increased substantially from 0.9% in 1980 to 11.6% in 2010 [10]. In 2016, the total number of people with diabetes in China reached 100 million and is growing steadily [11]. China is currently home to the largest number of people with diabetes in the world. Poor management of diabetes has also led to higher death rates in China [12]. In the United States of America (USA), despite a decrease in the prevalence of diabetes, there are thought to be around 30.3 million people with diabetes, and 84.1 million with a pre-diabetic condition [13]. Therefore, diabetes mellitus remains a major public health problem in both China and the USA, imposing considerable health and economic burdens on individuals and society [14,15].

There is lack of comparative research on NCDs in general, and on diabetes mellitus in particular, across developed and developing countries. This study aims to compare the main characteristics of research studies on diabetes conducted in China and the USA. The study aims to understand the similarities and differences in research designs, types, and themes in respect of diabetes in China and USA, and to provide empirical evidence reflecting public health research policies and priorities in China and the USA.

## 2. Methods

### 2.1. Inclusion and Exclusion Criteria

Eligible studies met the following inclusion criteria:

- Primary research studies or secondary analyses of routinely collected data or existing research datasets.
- Studies on diabetes research (e.g., risk factors, prognosis, prevention, diagnosis, treatment, and epidemiology).
- Studies conducted in mainland China or the USA.
- Studies published either in 2010 or 2015, in scientific journals, and with abstracts.

Studies were excluded if they were published in languages other than English or were literature reviews, editorials, letters, or study protocols. 

### 2.2. Study Search and Selection Strategy

**Figure 1:**
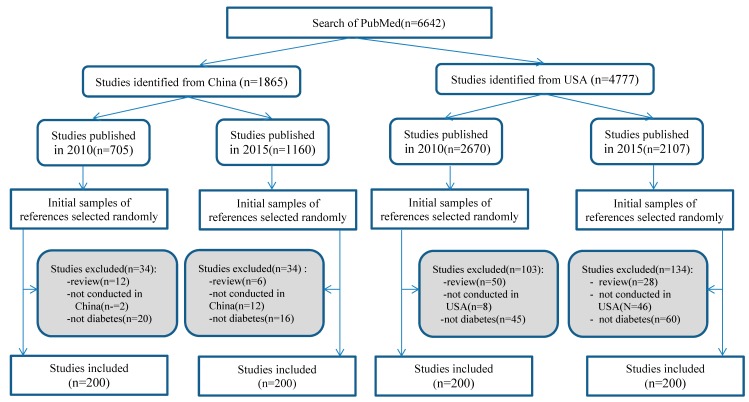
Selection process of relevant studies.

We searched PubMed to identify studies published either in 2010 or 2015, conducted in China or the USA. Studies were searched up to May 2016 using the search strategy described in supplementary. Citations of all identified records were downloaded to an EndNote database, and then exported to a Microsoft Excel spreadsheet by publication year and country. Each of the originally identified records by publication year and country was assigned a random number from 0 to 1 (generated by Excel). The records were listed from the smallest to the largest by the assigned random numbers. The first 200 eligible studies from each of the four groups (i.e., 2010 China, 2015 China, 2010 USA, and 2015 USA) were selected by screening titles and abstracts. If a selected study was not eligible, a successive record from the group was used to replace it until the total number of included studies was 200 for each of the four groups. Figure 1 shows the process of the selection of the 800 studies. 

### 2.3. Data Extraction and Analysis

Data extraction was completed in an Excel sheet (supplementary) using information from titles and abstracts of the included studies. The extracted data included information such as author, year of publication, country, language, study design, participants/subjects, relevance, risk factors investigated, and type of interventions. 

Pilot data extraction of the first 20 studies was conducted independently by two reviewers (HF and FS). The results were compared and any discrepancies discussed between the reviewers. The data extraction sheet was revised accordingly based on the results of the pilot. The remaining studies were extracted by two independent researchers (GJ and HF) and checked by two other independent researchers (FS and SB). Any disagreements were resolved through discussion. 

The main characteristics of the included studies are summarized in Table 1. The results were stratified by country. We compared the descriptive summary statistics for studies published in 2010 and 2015. The included data was processed and analyzed by Epidata 3.1 and SPSS 20.0 (SPSS, Inc., Chicago, IL, USA). 

## 3. Results

The PubMed search yielded a total of 6642 references for studies published in 2010 and 2015. The number of studies conducted in China was 705 in 2010 and 1160 in 2015, and the number of studies conducted in the USA was 2670 in 2010 and 2107 in 2015. Considering time and resource limitations, a total of 800 studies were included in the analysis. The process of selection of relevant studies is shown in Figure 1. 

### 3.1. Main Characteristics of Included Studies

None of the included studies in 2010 had authors from multiple countries. For studies published in 2015, the proportion of studies with authors from multiple countries was 6.5% in China and 24.5% in the USA. Studies from the USA were all published in English. The proportion of studies published in English in China increased over time (75.5% in 2010 versus 90.0% in 2015). Primary studies accounted for 96% of all included studies from China, and 75.7% from the USA. 

Studies from China were more likely to be laboratory-based (50.5% versus 30.8%), less likely to be clinical trials (6.2% versus 14.5%) or cohort studies (8.8% versus 26.0%), and more likely to be case-control studies (17.7% versus 10.0%), compared with those conducted in the USA. The proportion of randomized controlled trials (RCTs) in China remained very low in both 2010 (6%) and 2015 (5%). 

Compared to studies from the USA, studies from China, were less likely to include patients as subjects (42.7% versus 60.7%) and more likely to use animals as subjects (47.0% versus 27.5%). The majority of the included studies considered type 1 or 2 diabetes (88.3% in China and 85.7% in the USA). A small proportion of studies considered gestational diabetes (3% in China and 2% in the USA), and some studies considered diabetes with other diseases (e.g. chronic kidney disease, hypertension, HIV infection, etc.) (8.7% in China and 12.3% in the USA). 

### 3.2. Relevance of the Included Studies

Table 2 shows the relevance of the included studies. The proportion of studies relevant to treatments was higher in China (42.3%) than the USA (37.3%). Similar percentages were observed in both countries for diagnosis (7.7% in both countries) and prognosis (6.0% versus 7.5%). Studies in China were more likely to be relevant to disease risk factors (22.7%) compared to those in the USA (14.7%). The percentage of studies relevant to prevention was small, particularly in China (0.7% versus 3.3%). 

### 3.3. Type of Interventions

Table 3 shows the type of interventions investigated. Nearly half of the included studies evaluated interventions. Pharmacological interventions were evaluated more in China (36.5%) than in the USA (20.7%). However, studies evaluating disease management interventions were less common in China (3.3%) than in the USA (13.3%). Of all the included studies, only 0.2% in China evaluated behavioral interventions, compared with 5.8% in the USA.

## 4. Discussion

Existing evidence on the comparison of research on diabetes between developing and developed countries is limited. Diabetes is one of the diseases resulting in high morbidity and mortality in China, but the quantity of research carried out appears to be disproportionately small given the health and economic burden of disease. This comparative study of randomly selected studies from the USA and China highlights the difference in diabetes research between these countries. 

**Figure 2:**
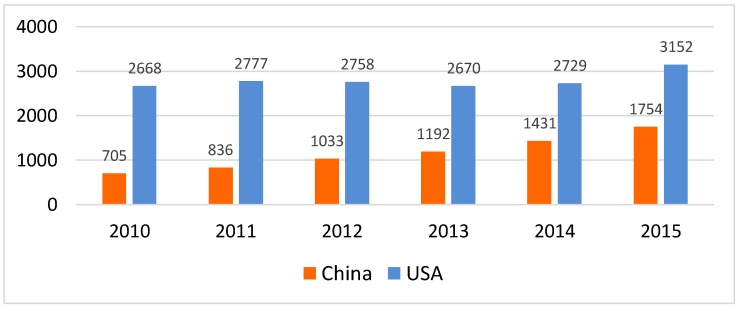
Number of published studies on diabetes research from China and the United States (Note: Based on a search of PubMed on 26/07/2017 using the search strategy in supplementary).

The total number of published studies on diabetes from China was lower compared to the USA (Figure 2). Several underlying factors may contribute to the lower number of diabetes studies in China. Historically, diabetes has not been a public health priority in developing countries [16]. In addition, diabetes research conducted in China may remain unpublished or may be published in national journals that have not been indexed in PubMed. The number of studies on diabetes in China has significantly increased by nearly 150% (705 in 2010 to 1754 in 2015), while the number of studies on diabetes from the USA increased only by 18% (2669 in 2010 to 3152 in 2015). This rapid rise in output between 2010 and 2015 indicates an increase in diabetes research in China, and also represents increased local activities within the country on the development and implementation of evidence-based solutions for diabetes.

Authorship of published research from multiple countries directs the international collaboration in the diabetes research field. The findings of this study show that international collaboration in diabetes research was previously limited in China compared with the USA. Our previous study showed that that research conducted in developed countries tends to beat a lower risk of bias [7]. Given this lower bias in developed countries, international research collaborations between developed and developing countries may encourage capacity building and quality improvement, and equally enhance care for patients with diabetes. Large multinational research that crosses cultures and levels of socioeconomic development might have wider applicability than single-country research [17]. To tackle the rising global burden of diabetes, more international collaboration between countries should be encouraged.

The current study shows that researchers from the USA were more likely to use secondary data compared to those in China. Health authorities and researchers worldwide have been collecting large amounts of primary data for decades. Previously, the utilization of such data has been very limited due to difficulties in access, scale, and linkages. There are growing efforts to reduce waste in research by using existing data fully to its capacity, especially in developed countries [18]. Routinely collected data might have the potential to facilitate research capable of providing answers to important health questions efficiently and cost-effectively. The key issue is whether such data can be reliably used to produce high quality research. With data access opening, secondary data should be considered before conducting any more primary studies [19]. In the past evidence obtained from analyzing secondary data was scarce. This gap has now been addressed by researchers [20]. 

The majority of diabetes studies both in the USA and China was laboratory-based research, and the percentage of laboratory-based research in China was higher than that in the USA (50.5% versus 30.8%). Compared with studies in the USA, studies in China were more likely to use animals as study subjects (47.0% versus 27.5%) and less likely to directly involve patients (42.7% versus 60.7%). In addition, studies in China was more likely to focus on disease risk factors than those in the USA (22.7% versus 14.7%).

The number of studies on treatment for diabetes was higher in China than in the USA (42.3% versus 37.3%). There were a small proportion of studies directly relevant to the prevention of diabetes in both countries (0.7% in China versus 3.3% in the USA). Regarding the type of interventions, studies in China were more likely to focus on pharmacological treatments (36.5% versus 20.7%) and less likely to study disease management interventions (3.3% versus 13.3%) or behavioral or educational interventions (0.2% versus 5.8%), compared with studies in the USA. RCTs provide the most valid evidence on the effects of clinical and public health interventions. However, the proportion of RCTs in China was much lower than that of the USA (5.5% versus 12.5%). 

The type of studies on diabetes reflects research priorities perceived by funding bodies and researchers. Although laboratory-based and animal studies are required to understand the pathological process and biological mechanisms of diabetes, translation of findings from such studies to clinical applications usually takes decades, and even highly promising basic research often fails to be clinically useful [21]. Patient-based clinical studies may generate findings that are more relevant to current health care practice for the prevention and management of diabetes. Therefore, balanced allocation of research funding on different types of research studies is required. Compared with studies in the USA, studies in China were more likely to be laboratory-based or animal studies, and less likely to be patient-based clinical trials. The Healthy China 2030 plan, issued by the Chinese government in 2016, lists of its key objectives as increasing the population’s life expectancy from 76 to 79 years by 2030 [22]. To achieve this objective, patient- and community-based studies are required to provide evidence on the prevention and treatment of diseases. Particularly, the number of RCTs and studies on disease management interventions in China needs to increase to provide research evidence that can have an immediate impact on patient management and population health. Moreover, collaboration between researchers in more and less developed countries should be promoted to overcome problems caused by limited research capacity in less developed countries. 

A limitation of the present study is that we included only research published in 2010 and 2015, and further studies are required to monitor any changes in the main characteristics of research on diabetes. Furthermore, the present study revealed considerable differences in research on diabetes between China and the USA. Further studies are required to investigate possible reasons for and causes of the observed differences. 

## 5. Conclusions

The number of studies on diabetes in China is rapidly increasing, but is still lower compared to the USA. Studies of diabetes in China were more likely to be basic or animal studies, and less likely to be patient-based clinical trials. For the control and management of diabetes, more resources should be allocated to patient-based clinical studies. Collaboration between researchers in developed and less developed countries should be encouraged. 

## Figures and Tables

**Table 1:** The main characteristics of included studies.

Countries of Authors	Published in 2010		Published in 2015		All		
	China	USA	China	USA	China	USA	*p*
China	200 (100%)	0 (0%)	187 (93.5%)	0 (0%)	387 (96.7%)	0 (0%)	0.000 *
USA	0 (0%)	200 (100%)	0 (0%)	151 (75.5%)	0 (0%)	351 (87.7%)	
Multiple	0 (0%)	0 (0%)	13 (6.5%)	49 (24.5%)	13 (3.3%)	49 (12.3%)	
Publication language							
Chinese	49 (24.5%)	0 (0%)	20 (10%)	0 (0%)	69 (17.3%)	0 (0%)	0.000 *
English	151 (75.5%)	200 (100%)	180 (90%)	200 (100%)	331 (82.7%)	400 (100%)	
Data source							
Primary	190 (95%)	139 (69.5%)	194 (97%)	164 (82%)	384 (96%)	303 (75.7%)	0.000 *
Secondary	10 (5%)	61 (30.5%)	6 (3%)	36 (18%)	16 (4%)	97 (24.3%)	
Design							
Case series	13 (6.5%)	5 (2.5%)	1 (0.5%)	3 (1.5%)	14 (3.5%)	8 (2.0%)	0.000 **
Case-control	39 (19.5%)	19 (9.5%)	32 (16%)	21 (10.5%)	71 (17.7%)	40 (10%)	
Clinical trial	14 (7%)	25 (12.5%)	11 (5.5%)	33 (16.5%)	25 (6.2%)	58 (14.5%)	
Cohort	12 (6%)	53 (26.5%)	23 (11.5%)	51 (25.5%)	35 (8.8%)	104 (26%)	
Cross sectional	24 (12%)	37 (18.5%)	29 (14.5%)	30 (15%)	53 (13.3%)	67 (16.7%)	
Laboratory-based	98 (49%)	61 (30.5%)	104 (52%)	62 (31%)	202 (50.5%)	123 (30.8%)	
Study subjects							
Animal	92 (46%)	55 (27.5%)	96 (48%)	55 (27.5%)	188 (47%)	110 (27.5%)	0.000 **
Bio-sample	6 (3%)	5 (2.5%)	8 (4%)	6 (3%)	14 (3.5%)	11 (2.7%)	
Care providers	0 (0%)	4 (2%)	0 (0%)	1 (0.5%)	0 (0%)	5 (1.3%)	
Patients	86 (43%)	120 (60%)	85 (42.5%)	123 (61.5%)	171 (42.7%)	243 (60.7%)	
Population	16 (8%)	12 (6%)	10 (5%)	14 (7%)	26 (6.5%)	26 (6.5%)	
Multiple	0 (0%)	3 (1.5%)	1 (0.5%)	1 (0.5%)	1 (0.3%)	4 (1%)	
Other	0 (0%)	1 (0.5%)	0 (0%)	0 (0%)	0 (0%)	1 (0.3%)	
Type of diabetes							
Type 1 and 2	191 (95.5%)	172 (86%)	162 (81%)	171 (85.5%)	353 (88.3%)	343 (85.7%)	0.221 *
Gestational	4 (2%)	4 (2%)	8 (4%)	4 (2%)	12 (3%)	8 (2%)	
With other diseases	5 (2.5%)	24 (12%)	30 (15%)	25 (12.5%)	35 (8.7%)	49 (12.3%)	

Notes: * *p* value is based on Pearson’s chi-square test. ** *p* value is based on Fisher’s exact test.

**Table 2:** Relevance evaluated in the included studies.

Relevance of the Included Studies	Published in 2010		Published in 2015		All		
	China	USA	China	USA	China	USA	*p* Value
Consequence	0 (0%)	2 (1%)	0 (0%)	5 (2.5%)	0 (0%)	7 (1.7%)	0.015 **
Diagnosis	19 (9.5%)	15 (7.5%)	12 (6%)	16 (8%)	31 (7.7%)	31 (7.7%)	1.000 *
Disease risk factors	47 (23.5%)	29 (14.5%)	44 (22%)	30 (15%)	91 (22.7%)	59 (14.7%)	0.005 *
Epidemiology	6 (3%)	9 (4.5%)	3 (1.5%)	7 (3.5%)	9 (2.3%)	16 (4%)	0.222 *
Prevention	1 (0.5%)	7 (3.5%)	2 (1%)	6 (3%)	3 (0.7%)	13 (3.3%)	0.020 *
Prognosis	10 (5%)	14 (7%)	14 (7%)	16 (8%)	24 (6%)	30 (7.5%)	0.481 *
Treatment	84 (42%)	79 (39.5%)	85 (42.5%)	70 (35%)	169 (42.3%)	149 (37.3%)	0.170 *
Multiple	2 (1%)	0 (0%)	3 (1.5%)	3 (1.5%)	5 (1.3%)	3 (0.7%)	0.725 **
Other	31 (15.5%)	45 (22.5%)	37 (18.5%)	47 (23.5%)	68 (17%)	92 (23%)	0.042 *

Notes: * *p* value is based on Pearson’s chi-square test; ** *p* value is based on Fisher’s exact test. Other: including pathology, physiology, or diabetes as a risk factor for other diseases, etc.

**Table 3:** Interventions evaluated in the included studies.

Type of Interventions	Published in 2010		Published in 2015		all		
	China	USA	China	USA	China	USA	*p*
Behavioral or Educational	0 (0%)	12 (6.0%)	1 (0.5%)	11 (5.5%)	1 (0.2%)	23 (5.8%)	0.000 *
Biological	8 (4%)	5 (2.5%)	6 (3%)	7 (3.5%)	14 (3.5%)	12 (3%)	0.842 *
Diagnostic tests	10 (5%)	11 (5.5%)	12 (6%)	11 (5.5%)	22 (5.5%)	22 (5.5%)	1.000 *
Disease management	8 (4%)	34 (17%)	5 (2.5%)	19 (9.5%)	13 (3.3%)	53 (13.3%)	0.000 *
Pharmacological	71 (35.5%)	41 (20.5%)	75 (37.5%)	42 (21%)	146 (36.5%)	83 (20.7%)	0.000 *
Radiological	1 (0.5%)	0 (0%)	0 (0%)	0 (0%)	1 (0.3%)	0 (0%)	1.000 **
Surgical	4 (2%)	10 (5%)	5 (2.5%)	3 (1.5%)	9 (2.3%)	13 (3.3%)	0.518 *
Multiple	3 (1.5%)	1 (0.5%)	0 (0%)	2 (1%)	3 (0.7%)	3 (0.7%)	1.000 **
NA	95 (47.5%)	83 (41.5%)	93 (46.5%)	101 (50.5%)	188 (47%)	184 (46%)	0.557 *
Other	0 (0%)	3 (1.5%)	3 (1.5%)	4 (2%)	3 (0.7%)	7 (1.7%)	0.341 *

Notes: * *p* value is based on Pearson’s chi-square test; ** *p* value is based on Fisher’s exact test.

## Supplementary Materials

The following are available online at https://www.mdpi.com/1660-4601/16/16/2989/s1, Figure S1: title, Table S1: title, Video S1: title.

## Abbreviations

NCDs	Non-communicable diseases
LMICs	Low and Middle Income Countries
USA	United States of America
RCTs	Randomized Controlled Trials
