# Supplementary material for: Differences and Similarities in Diabetes Research between China and the USA
Source: Int J Environ Res Public Health. 2019 Aug 20;16(16):2989. doi: 10.3390/ijerph16162989 (PMC6720953; doi:10.3390/ijerph16162989)
Supplement: Supplementary file 1 [file ijerph-16-02989-s001.pdf]

Appendix1: Protocol -comparison of diabetes studies in China and USA
Scoping review Protocol

Study inclusion criteria

Eligible studies should meet all of the following inclusion criteria:

- Primary research studies or secondary analyses of routinely collected data or existing research datasets
- Studies on diabetes (such as causes, risk factors, prognosis, prevention, diagnosis, treatment, epidemiology, and so on)
- Studies conducted in mainland China or USA
- Studies published in 2010 and 2015, in journals and with abstracts.

We excluded literature review based articles, editorials, letters, and study protocols.

Study search and selection

We will search PubMed to identify relevant studies conducted in mainland China and USA, and published in 2010 and 2015, using the search strategy below:

> China[Affiliation] AND diabetes[mh] NOT (systematic review[Title] OR meta-analysis[Title] OR "REVIEW"[ptyp] OR editorial[ptyp] OR comment[ptyp] OR letter[ptyp] OR protocol[Title])) AND hasabstract[text] AND "Journal Article"[ptyp] AND ("2010/01/01"[Date - Publication] : "2015/12/31"[Date - Publication])

> (US[Affiliation] OR USA[Affiliation] OR United States[Affiliation] OR Alabama[Affiliation] OR Alaska[Affiliation] OR Arizona[Affiliation] OR Arkansas[Affiliation] OR California[Affiliation] OR Colorado[Affiliation] OR Connecticut[Affiliation] OR Delaware[Affiliation] OR Florida[Affiliation] OR Georgia[Affiliation] OR Hawaii[Affiliation] OR Idaho[Affiliation] OR Illinois[Affiliation] OR Indiana[Affiliation] OR Iowa[Affiliation] OR Kansas[Affiliation] OR Kentucky[Affiliation] OR Louisiana[Affiliation] OR Maine[Affiliation] OR Maryland[Affiliation] OR Massachusetts[Affiliation] OR Michigan[Affiliation] OR Minnesota[Affiliation] OR Mississippi[Affiliation] OR Missouri[Affiliation] OR Montana[Affiliation] OR Nebraska[Affiliation] OR Nevada[Affiliation] OR "New Hampshire"[Affiliation] OR "New jersey"[Affiliation] OR "New mexico"[Affiliation] OR "New York"[Affiliation] OR "North Carolina"[Affiliation] OR "North Dakota"[Affiliation] OR Ohio[Affiliation] OR Oklahoma[Affiliation] OR Oregon[Affiliation] OR Pennsylvania[Affiliation] OR "Rhode island"[Affiliation] OR "South Carolina"[Affiliation] OR "South Dakota"[Affiliation] OR Tennessee[Affiliation] OR Texas[Affiliation] OR Utah[Affiliation] OR Vermont[Affiliation] OR Virginia[Affiliation] OR Washington[Affiliation] OR West Virginia[Affiliation] OR Wisconsin[Affiliation] OR Wyoming[Affiliation]) AND diabetes[mh] NOT (systematic

review[Title] OR meta-analysis[Title] OR "REVIEW"[ptyp] OR editorial[ptyp] OR comment[ptyp] OR letter[ptyp] OR protocol[Title]) AND hasabstract[text] AND "Journal Article"[ptyp] AND ("2010/01/01"[Date - Publication] : "2015/12/31"[Date - Publication])

We will randomly select a total of 800 studies from records identified by search PubMed:

	China	USA
2010	200	200
2015	200	200
Total	400	400

Data extraction and analysis

We will extract data based on information available from titles and abstracts of included studies into an Excel sheet. Appendix 2 shows data extraction and coding methods. Data extracted will include: author, year of publication, country, language, study design, participants/subjects, relevance, risk factors investigated, and type of interventions.

Data extraction of the first 20 studies will be conducted independently by two reviewers (FH, SB or FS). We will then compare data extracted, and discuss any disagreements. The data extraction sheet will be revised according to results of the pilot testing. If necessary, we will test the revised data extraction and coding methods using 20 more studies, until the agreement between reviewers is satisfactory. One reviewer will extract and a second reviewer will check data from remaining studies.

Data extraction and coding (based on titles/abstracts)

Note to Appendix 1: (1) Study design coding is based on: Grimes and Schulz. An overview of clinical research: the lay of the land. Lancet 2002, 359: 57-61.

Heading	Coding	Details
**Endnote number**	#	
**Include**	Yes	Initially selected studies may be excluded according to inclusion/exclusion criteria
	No	
**First author name**		
**Year of publication**		
**Journal**	Journal name	
**Country**	China	In which data collection or experiments were conducted
	USA	
**Authors from multiple countries**	No	
	Yes	If Yes, list the name of other countries
**Language**	English	
	Chinese	
	Other: __________	
**Data source**	Primary	Primary data collected, including multiple publications of the same study
	Secondary	Used existing datasets, including routinely collected data or existing research datasets.
**Design** (1)	Laboratory-based	Animal studies; and studies based on tissues, cells, and other bio-samples, without human participants involved.
	Clinical trial -RCT	Experimental study -Randomised controlled trials that evaluated effects or adverse effects of healthcare interventions.
	Clinical trial -NRS	Experimental study -Non-randomised clinical trials; including historically controlled trials
	Case series	Retrospective, observational case reports, sample size was usually small (n<100)
	Cohort-prospective	Prospective observational studies with comparison group: Exposure -> Outcome
	Cohort-retrospective	Retrospective observational studies with comparison group (n>=100)
	Case Control	Observational studies with comparison group: Outcome -> Exposure
	Cross sectional	Observational studies: Exposure & Outcome at the same time
	Other	Please specify
**Subjects**	Animal	Please specify species
	Bio-sample	E.g, human tissues, cancer cells, blood samples
	Patients	People with clinically diagnosed conditions
	Population	General population in communities
	Care providers	E.g., nurses, physicians, decision makers
	Other	Please specify. E.g., air, water, and other environmental samples
**NCD category**	Diabetes	Types: Type I, Type II, Pre-diabetic
	Multiple	Diabetes and other conditions
**Relevance**	Disease risk factors	Risk factors that were associated with diabetes
	Prognosis	
	Diagnosis	Including screening, and monitoring of conditions
	Treatment	
	Prevention	
	Epidemiology	E.g., disease distribution, trends

Heading	Coding	Details
	Consequence	For example, impact of diabetes on cost, economics
	Multiple	List relevant items
	Other	Please specify; Including diabetes as a risk factor for other diseases (such as stroke)
**Risk factors**	Tobacco	
	Alcohol	
	Inactivity	
	Dietary	
	Body weight	
	Blood pressure	
	Biomarker	Details: such as cholesterol, blood sugar, etc.
	Genetic	
	Medical	Adverse effects of medications or other treatments
	Environmental	Natural factors, e.g., air, water, climate
	Demographic	E.g, age, sex, ethnicity
	Socioeconomic	Social, economic factors
	Multiple	List multiple factors
	Other	Please specify
	NA	Not applicable
**Interventions**	Diagnostic tests	
	Pharmacological	
	Radiological	
	Surgical	
	Biological	e.g, cell therapy, biological products
	Educational/counselling	
	Behavioural	
	Disease management	E.g, implementation of guidelines, care pathways.
	Public health policy	e.g. ban smoking in public places
	Alternative/traditional	Specify when possible
	Other:	Please specify
	NA	Not applicable

Data analysis methods

The main study characteristics of the included will be descriptively summarised in tables.

Differences in study characteristics between China and USA will be compared, and statistically tested using Chi-square test. Statistical significance was defined as $P<0.05$.
